# Characterization of the *WRKY* gene family reveals its contribution to the adaptability of almond (*Prunus dulcis*)

**DOI:** 10.7717/peerj.13491

**Published:** 2022-07-04

**Authors:** Zhenfan Yu, Dongdong Zhang, Bin Zeng, Xingyue Liu, Jiahui Yang, Wenwen Gao, Xintong Ma

**Affiliations:** 1 College of Horticulture, Xinjiang Agriculture University, Urumqi, China; 2 GuangZhou Institute of Forestry and Landscape Architecture, GuangZhou, China

**Keywords:** Almond (*Prunus dulcis*), WRKY transcription factors, Genome-wide, Evolutionary analyses, Expression patterns, Abiotic stress

## Abstract

**Background:**

WRKY (WRKY DNA-binding domain) transcription factors an important gene family that widely regulates plant resistance to biological and abiotic stresses, such as drought, salt and ion stresses. However, research on the *WRKY* family in almond has not yet been reported. Almond is an economically important fruit tree in Xinjiang that have strong resistance to various stresses.

**Results:**

A total of 62 *PdWRKY* genes were identified (including six pairs of homologous genes), and the phylogenetic tree was divided into three groups according to the WRKY domain and zinc finger motifs. The members of each group had a significant number of conserved motifs and exons/introns distributed unevenly across eight chromosomes, as well as 24 pairs of fragment duplicates and nine pairs of tandem duplicates. Moreover, the synteny and Ka/Ks analyses of the *WRKY* genes among almond and distinct species provided more detailed evidence for *PdWRKY* genes evolution. The examination of different tissue expression patterns showed that *PdWRKY* genes have tissue-specific expression characteristics. The qRT–PCR results showed that *PdWRKY* genes participate in the resistance of almond to the effects of low-temperature, drought and salt stress and that the expression levels of these genes change over time, exhibiting spatiotemporal expression characteristics. It is worth noting that many genes play a significant role in low-temperature stress resistance. In addition, based on the conserved WRKY motif, 321 candidate target genes were identified as having functions in multiple pathways.

**Conclusions:**

We conducted systematic bioinformatics analysis and abiotic stress research on the *WRKY* gene family in almond, laying the foundation for future *PdWRKY* genes research and improvements to almond production and breeding.

## Introduction

WRKY transcription factors bind to the specific promoter sequences (W-box) of target genes and can positively or negatively regulate target genes expression (Ulker & Somssich, 2004). The *WRKY* gene family is one of the largest and most widely studied transcription factor (TF) families in higher plants. Since the first *WRKY* gene was cloned in sweet potato (Ishiguro & Nakamura, 1994), *WRKY* genes have been identified in various plants, including *Arabidopsis thaliana* (Dong, Chen & Chen, 2003), *Oryza sativa* (Ross, Liu & Shen, 2007), *Zea mays* (Zhang et al., 2017), *Prunus mume* (Bao et al., 2019), and *Ziziphus jujuba* (Xue et al., 2019). The WRKY protein has one or two 60 amino acid-long DNA-binding domains, including the conserved heptapeptide WRKYGQK, and two different zinc finger motifs, C2H2 (CX4-5CX22-23HXH) and C2HC (CX7CX23-24 HXC) (Eulgem et al., 2000). According to the number of WRKY domains and the specific zinc finger motif, WRKY-TFs can be divided into three phylogenetically different groups. Group I WRKYs have two WRKY domains, and Group II WRKYs contain one WRKY domain; Group I and Group II WRKYs both contain a C2H2-type zinc finger motif (C-X4-5-C-X22-23-H-X1-H). The third group has a WRKY domain and a C2HC-type motif (C-X7-C-X23-H-X1-C). In addition, on the basis of phylogenetic analysis, Group II is further divided into five subgroups (IIa, IIb, IIc, IId, and IIe) (Wu et al., 2005; Muthamilarasan et al., 2015; Mohanta, Park & Bae, 2016). *WRKY* family members are important TFs with multiple functions that are involved in plant growth and development (Lagace & Matton, 2004), leaf senescence (Miao et al., 2004), flowering (Li, Wang & Yu, 2016), fruit and pollen development (Guan et al., 2014), biological stress (Mukhtar et al., 2008), abiotic stress (Bao et al., 2018), and hormone signalling (Nan & Gao, 2019).

Studies have shown that many *WRKY* genes are involved in various stress responses. *AtWRKY70* is required for R gene-mediated pathogen responses and determines the balance between salicylic acid (SA)- and jasmonic acid (JA)-dependent defence systems (Li et al., 2006; Knoth et al., 2007). *AtWRKY38* and *AtWRKY62* are negative regulators of basal resistance to bacterial pathogens (Kim et al., 2008). *AtWRKY6* and *AtWRKY42* participates in the response to low stress by regulating *PHO1* expression (Chen et al., 2009). In *Pennisetum glucum*, *PgWRKY33*, *PgWRKY62* and *PgWRKY65* are involved in dehydration and salt stress responses (Jeky et al., 2020). In *Vitis vinifera*, *VvWRKY30* was proven to convey salt tolerance (Zhu et al., 2019). Ten *OsWRKY* genes were found to be downregulated or upregulated under salt, drought, cold and heat stresses in rice. In summary, WRKY transcription factors may be involved in a variety of pathways, leading to a range of physiological responses. The evolution and relief expansion of *WRKY* genes seem to be related to their functional diversity (Rinerson et al., 2015).

Almond (*Prunus dulcis*) are one of the most important dried fruit foods in the world, and almond trees are also economically important economic fruit trees in Xinjiang, China. Therefore, this study used the whole-genome data of almond to analyse the protein characteristics, phylogenetic relations, motifs, exon/intron relationships, gene locations, gene duplication types, Ka/Ks ratios and expression patterns of *WRKY* gene family members. In addition, 10 *PdWRKYs* were further selected for qRT–PCR analysis. In general, this study provides insights for use in future functional studies of the *WRKY* gene in almond, which may be valuable for almond breeding.

## Materials and Methods

### Screening of *WRKY* gene family members

The GDR database (https://www.rosaceae.org/) was accessed to download the genome data for *Prunus dulcis* cv Texas (Alioto et al., 2020). The WRKY domain hidden Markov model (PF03106) dataset was downloaded from the Pfam database (Sonnhammer et al., 1998). The whole-genome protein data of *Prunus dulcis* were searched and compared with regard to the WRKY domain by using the Hmmer tool, and the E-value was set to ≤1e^−5^ (Finn, Clements & Eddy, 2011). The protein molecular weight, isoelectric point, and hydrophobicity of the protein were assessed. ExPASy (http://au.expasy.org/tools) and Wolfe PSORT II (https://www.genscript.com/wolf-psort.html?src=leftbar) were used for protein subcellular localization analysis (Gasteiger et al., 2003; Paul, Keun & Takeshi, 2007).

### Multiple sequence alignment and phylogenetic tree construction

Cluster Omega (https://www.ebi.ac.uk/Tools/msa/clustalo/) ClusterW multiple sequence alignment was performed on PdWRKY protein sequences (Sievers & Higgins, 2018). The *Arabidopsis thaliana* AtWRKY protein sequences were downloaded from the UniProt protein database (https://www.uniprot.org/). Multiple alignments of PdWRKY and AtWRKY proteins were performed using the MEGA X tool selection MUSCLE method (Kumar et al., 2018; Liu et al., 2018). The aligned sequences were analysed by the neighbour-joining (NJ) method to build a phylogenetic tree based on the following parameters: Poisson model, pairwise deletion, and 1,000 bootstrap replications.

### Analysis of the motifs and gene structures of *PdWRKY* family members

The MEME (https://meme-suite.org/meme/tools/meme) tool was used to perform conserved motif analysis on the PdWRKY protein sequences. The number of search motifs was set to 10, and the remaining parameters were set to the default values (Bailey, Mikael & Buske, 2009). The numbers of exons/introns in the *PdWRKY* gene sequences across the whole genome were extracted for gene structures analysis. Tbtools was used to combine the conserved protein motifs and gene structures with the *PdWRKYs* phylogenetic tree (Chen, Chen & Zhang, 2020).

### Chromosome location, collinearity and Ka/Ks analysis of the *PdWRKY* genes

According to the chromosome location information for the *PdWRKY* genes, TBtools software was used to draw the location distribution map (Chen, Chen & Zhang, 2020). MCScanX was used to analyse the collinearity of the *PdWRKY* genes (Wang, Tang & Debarry, 2012), and the Circos tool was used to produce a chromosome collinearity map (Martin et al., 2009). In addition, we further selected *PdWRKY* and *WRKY* gene family members from *Arabidopsis thaliana* and *Rosaceae* plants (*Malus domestica*, *Prunus persica*, *Prunus avium*, *Prunus armeniaca* and *Rosa chinensis*) to study the collinear gene distribution relationship, and we used the Ka/Ks Calculator tool to calculate the Ka/Ks ratios of the collinear genes (Wang, Zhang & Zhang, 2010).

### Analysis of *cis*-elements in *PdWRKY* genes

The 2,000-bp upstream sequences of the identified *PdWRKY1* genes were extracted using TBtools. The extracted sequences were then submitted to the PlantCARE website (http://bioinformatics.psb.ugent.be/webtools/plantcare/html/) to predict the *cis*-elements in the promoter regions (Lescot, 2002). A diagram of the *cis*-elements within the *PdWRKY1* genes was constructed using TBtools and modified *via* Adobe Illustrator CC 2019 (Chen, Chen & Zhang, 2020), and statistical analyses were performed (Liu et al., 2018).

### Expression pattern analysis

Transcriptome data from four almond tissues (leaves, buds, flowers, and pistils) were downloaded from the NCBI SRA database to obtain the corresponding FPKM values, and the expression patterns of *PdWRKY* genes in different tissues were analysed.

### Material handling and qRT–PCR analysis

To analyse the transcription level of each *PdWRKY* genes after different abiotic stress treatments, 1-year-old ‘Zhipi’ almond seedlings were collected from the almond resource nursery in Shache County, Xinjiang. These seedlings were placed in Murashige and Skoog (MS) liquid medium containing 300 mmol/L NaCl and 20% polyethylene glycol (PEG; mass fraction) and placed in an artificial climate chamber at 4 °C to simulate low-temperature stress (Alam et al., 2019). For each treatment, the leaves were collected at 0, 6, 12 and 24 h.

To verify the expression pattern of 1-year-old almond seedlings, we selected 10 representative *PdWRKY* genes for qRT–PCR analysis. Three independent biological repeats containing three independent plants were used for qRT–PCR detection. The qRT–PCR primers of the selected *PdWRKY* genes were designed by Primer Premier 5 (Table S1). Total RNA was extracted from frozen embryonic samples by using the RNAprep Pure Plant Kit (TIANGEN, Beijing, China). The reaction system (25 μL) consisted of the following: 10 μL of SYBR Green PCR Master Mix, 0.4 μL of forward primer (10 μmol/L), 0.4 μL of reverse primer (10 μmol/L), 5 μL of diluted cDNA (50 ng/μL), and 25 μL of RNase-free water. The reaction conditions were as follows: predenaturation at 95 °C for 3 min; 40 cycles of 95 °C for 10 s, 55 °C for 20 s, and 72 °C for 20 s; and finally, 75 °C for 5 s. Plate reads were then performed to detect fluorescence signals (40 cycles). Melting curve analysis was conducted at a temperature range of 65–95 °C with a temperature increment of 0.5 °C/5 s. The obtained cycle threshold (CT) values were quantitatively analysed by the 2^−ΔΔCT^ method (Livak & Schmittgen, 2001) (Table S2).

### Construction of the PdWRKY proteins interaction network

The PdWRKY protein sequences were uploaded to the STRING database (https://string-db.org/) for comparison, and the relationships between important proteins were predicted based on *Arabidopsis thaliana* protein interactions. Gene Ontology (GO) annotation of *PdWRKY* genes was performed using TBtools.

### Identification and annotation of target genes of *PdWRKY* genes

To obtain the possible downstream target genes regulated by *WRKY*, TBtools was used to extract the 2,000-bp gene promoter sequences for almond genes. The conserved motif of the WRKY DNA-binding site (MA1298.1) was obtained from the JASPA_CORE database, which contains eukaryotic TF binding profiles (http://jaspar.genereg.net) (Aziz et al., 2018). Then, the Motif FIMO (https://meme-suite.org/meme/) tool was used to detect the motifs in the almond promoters that bound to *WRKY*. The final candidate target genes were determined based on the screening criteria of *P* < 1 × e^−6^ (Bailey, Mikael & Buske, 2009). Target genes of candidate *WRKYs* were functionally annotated using GO and the Kyoto Encyclopedia of Genes and Genomes (KEGG) databases. Analysis and visualization were performed using the Omicshare online platform (https://www.omicshare.com/tools/). Finally, protein domain annotation of candidate target genes was performed with the InterPro database (https://www.ebi.ac.uk/interpro/).

## Results

### Analysis of the characteristics of *PdWRKY* family members

A total of 62 *WRKY* genes were screened from the whole genome of almond and renamed PdWRKY1–PdWRKY56. Six pairs of homologous genes were renamed *PdWRKY7a/7b*, *PdWRKY11a/11b*, *PdWRKY28a/28b*, *PdWRKY311a/31b*, *PdWRKY38a/38b* and *PdWRKY48a/48b*. The protein lengths of the PdWRKY family members ranged from 170 aa (PdWRKY40) to 748 aa (PdWRKY42); the molecular weight ranged from 19.55 kDa (PdWRKY40) to 80.85 kDa (PdWRKY42); and the isoelectric point ranged from 4.99 (PdWRKY38a) to 10.03 (PdWRKY13). The PdWRKY proteins GRAVY (grand average of hydropathicity) values ranged from −0.532 (PdWRKY13) to −1.096 (PdWRKY25), indicating hydrophilicity. The predicted results of subcellular localization indicated that all examined proteins were located in the nucleus (Table S3).

### Multiple sequence alignments results and phylogenetic relationships of *PdWRKY* family members

**Figure 1:**
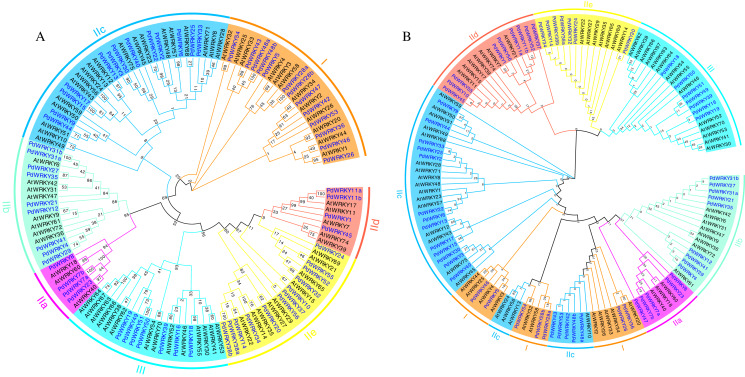
Almond and *Arabidopsis WRKY* member protein phylogenetic tree. (A) Neighbor-joining (NJ) phylogenetic tree. (B) Maximum likelihood (ML) phylogenetic tree. By using MEGA X, the multiple protein sequences in two species were aligned with the MUSCLE method, and the tree was built used the maximum likelihood (ML) method with the best scoring JTT + G + I model. Each colour represents a group, the blue font represents PdWRKY, the black font represents AtWRKY.

Almond *PdWRKY* family members are divided into three groups, I, II and III, according to the domains found in *AtWRKY* family members (Fig. 1). Among them, 12 *PdWRKYs* were distributed in Group I, seven *PdWRKYs* were distributed in Group III, and 43 *PdWRKYs* were distributed in Group II. In addition, Group II was further divided into five subgroups, IIa, IIb, IIc, IId and IIe, with four, nine, 14, nine and seven *PdWRKYs*, respectively. In addition, we further constructed a maximum likelihood (ML) phylogenetic tree. Compared to the neighbour-joining (NJ) phylogenetic tree, the number of *PdWRKYs* in group I was reduced to seven and the number of *PdWRKYs* in group II was increased to 48. Also, members of group I and subgroup IIc did not cluster on the same branch. Notably, the five genes in group I were *PdWRKY42*/*43*/*53*/*48a*/*48b* and they were divided into subgroup IIc. Therefore, the clustering results of the neighbour-joining phylogenetic tree were better. The WRKYGQK heptapeptide is considered a hallmark of the WRKY protein. The multiple alignment method was used to analyse the conservation of domains across PdWRKYs. The PdWRKYs all contained WRKYGQK and zinc finger domains (Fig. S1). The members of Group I all had two WRKYGQK domains, while those of Group II and Group III had only one WRKYGQK domain, and all examined proteins had conserved C-C and H-H/C domains. Additionally, PdWRKY7 and PdWRKY37 had a single amino acid substitution: K changed to Q.

### Motifs and gene structures of *PdWRKY* family members

**Figure 2:**
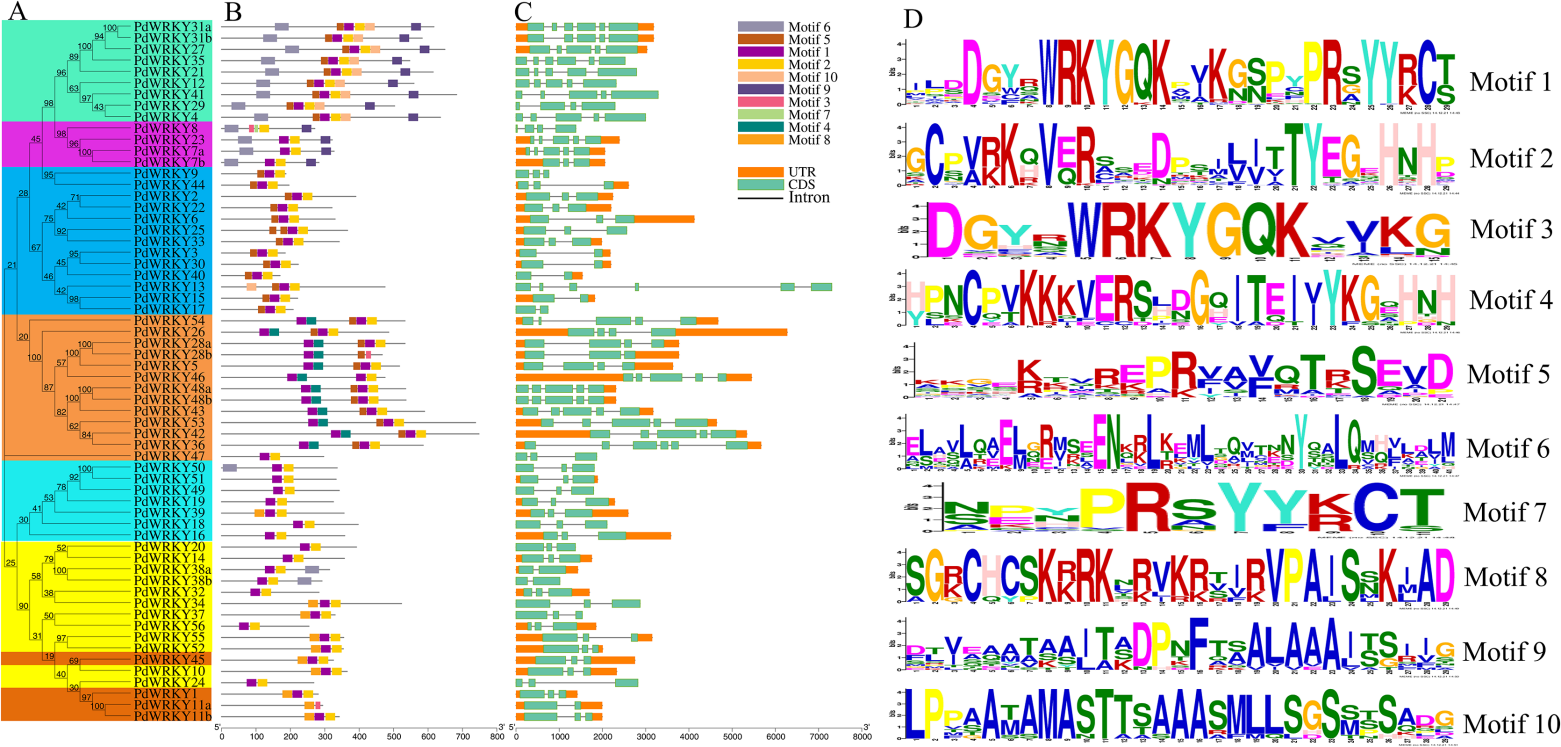
*PdWRKY* members Motifs and gene structures phylogenetic clustering. (A) Neighbor-joining phylogenetic tree of members of the PdWRKY family. Different coloured areas represent different groups. (B) Conserved protein motifs. (C) Gene structure. (D) Motif LOGO.

PdWRKY proteins have different motif types (Fig. 2B). Motifs 1 and 2 are typical WRKY domains (Fig. 2D). All PdWRKY proteins contain Motif 1, except for PdWRKY8. Moreover, the motif types were conserved within the same group, indicating that members in the same group have similar functions. Members in Group I all have two copies of Motif 1, which is consistent with the multiple sequence alignments results.

To explore the gene structures of *PdWRKYs*, we constructed an exon/intron structures map (Fig. 2C). The results showed that *PdWRKYs* have at least one intron, and four, 15, five, 30 and eight members have six, five, four, three and two exons, respectively. The genes in the same group have similar structures; for example, all genes in Group III have three exons and two introns. In addition, we compared the distribution of *WRKY* sequences and exons for each *PdWRKY* and found that most *PdWRKYs* have *WRKY* domains covering or spanning one exon, Members in Group I have two *WRKY* domains, often spanning two exons.

### Gene mapping, collinearity and Ka/Ks of *PdWRKY* family members

**Figure 3:**
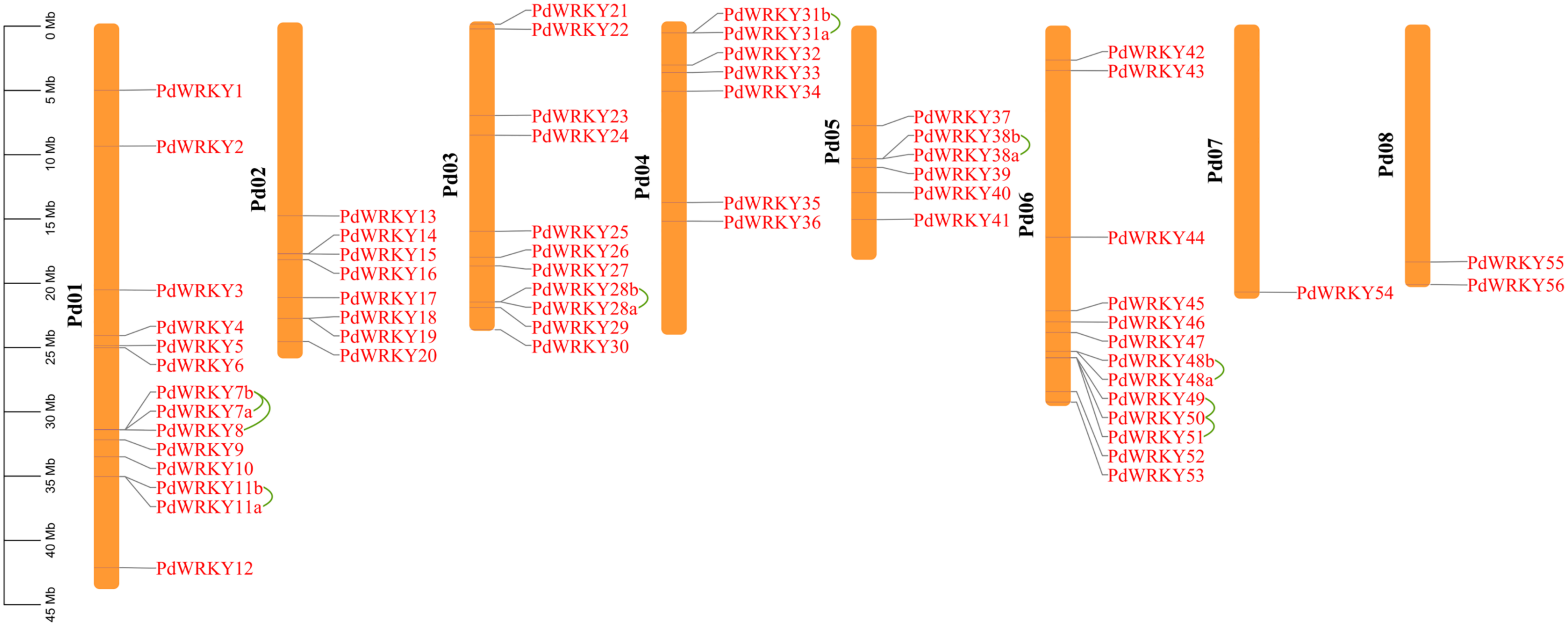
Chromosome localization of almond *WRKY* family members. Green lines indicate tandem repeat gene pairs.

The gene chromosome mapping results showed that 62 *PdWRKY* members were unevenly distributed on eight chromosomes (Fig. 3). Chromosomes 1–8 contained 14 (22.58%), 8 (12.90%), 11 (17.4%), 7 (11.29%), 6 (9.68%), 13 (20.97%), 1 (1.61%) and 2 (3.2%) members. These results indicate that *PdWRKY* genes can mainly be found on chromosomes 1–6.

**Figure 4:**
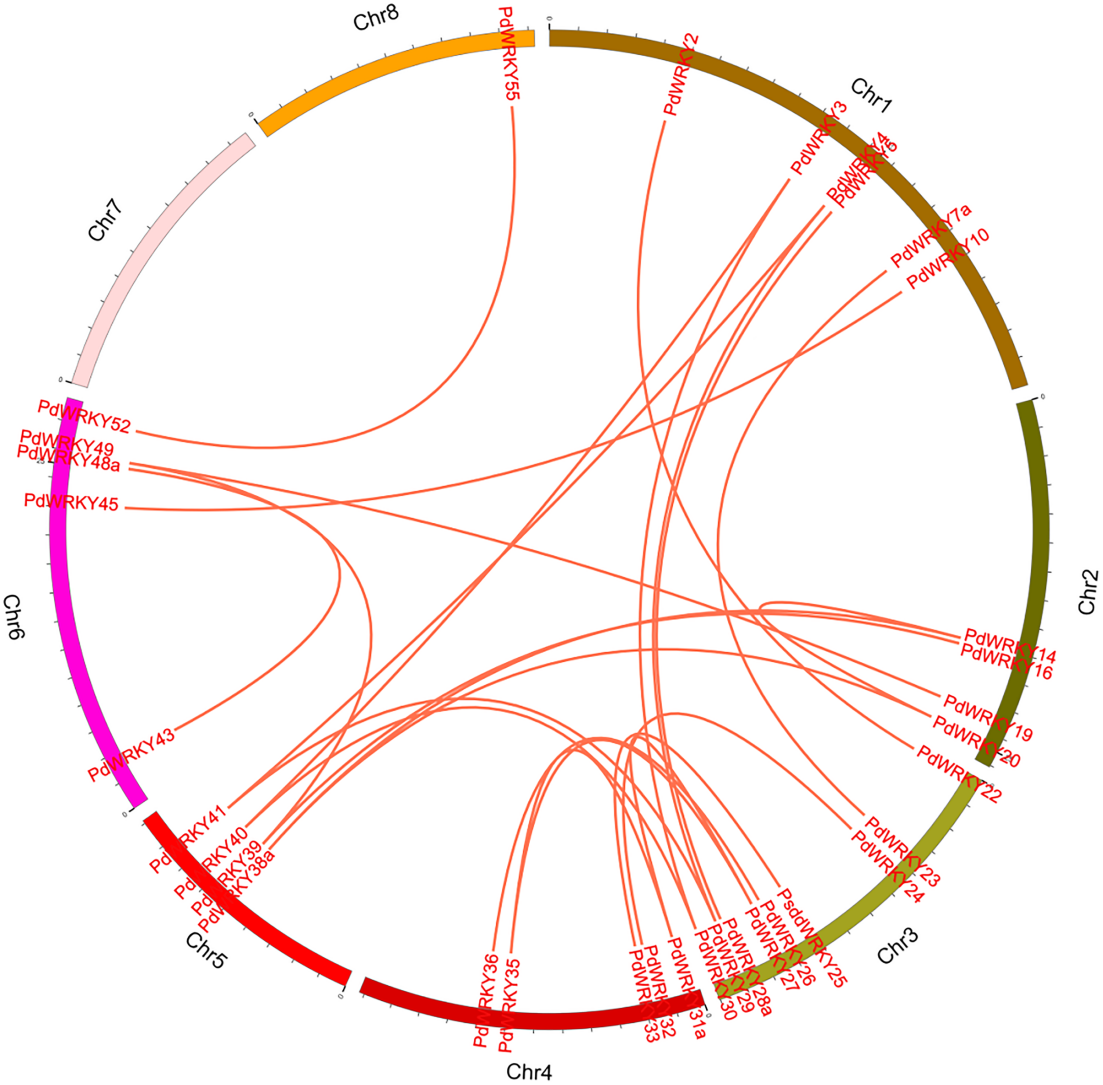
Inter-chromosomal relations of the *PdWRKY* genes in the almond genome. The red lines linked the duplicated *PdWRKY* gene pairs.

The collinearity results for genes within the genome showed that the *PdWRKYs* had 24 pairs of segmental duplications (Fig. 4) and nine pairs of tandem replications (Fig. 3; Table S4). The segmental duplications were mainly distributed on six chromosomes: Chr1 (six), Chr2 (four), Chr3 (nine), Chr4 (five), Chr5 (four) and Chr6 (five). Chr8 has only one *PdWRKY* gene, *PdWRKY55*, Chr7 has no *PdWRKY* gene, and most of the segmental duplications of *PdWRKY* genes occur in Group II. There were two or more pairs of tandem repeats in Groups I, II and III, which were mainly distributed on chromosomes 1 and 6 (Fig. 3).

**Figure 5:**
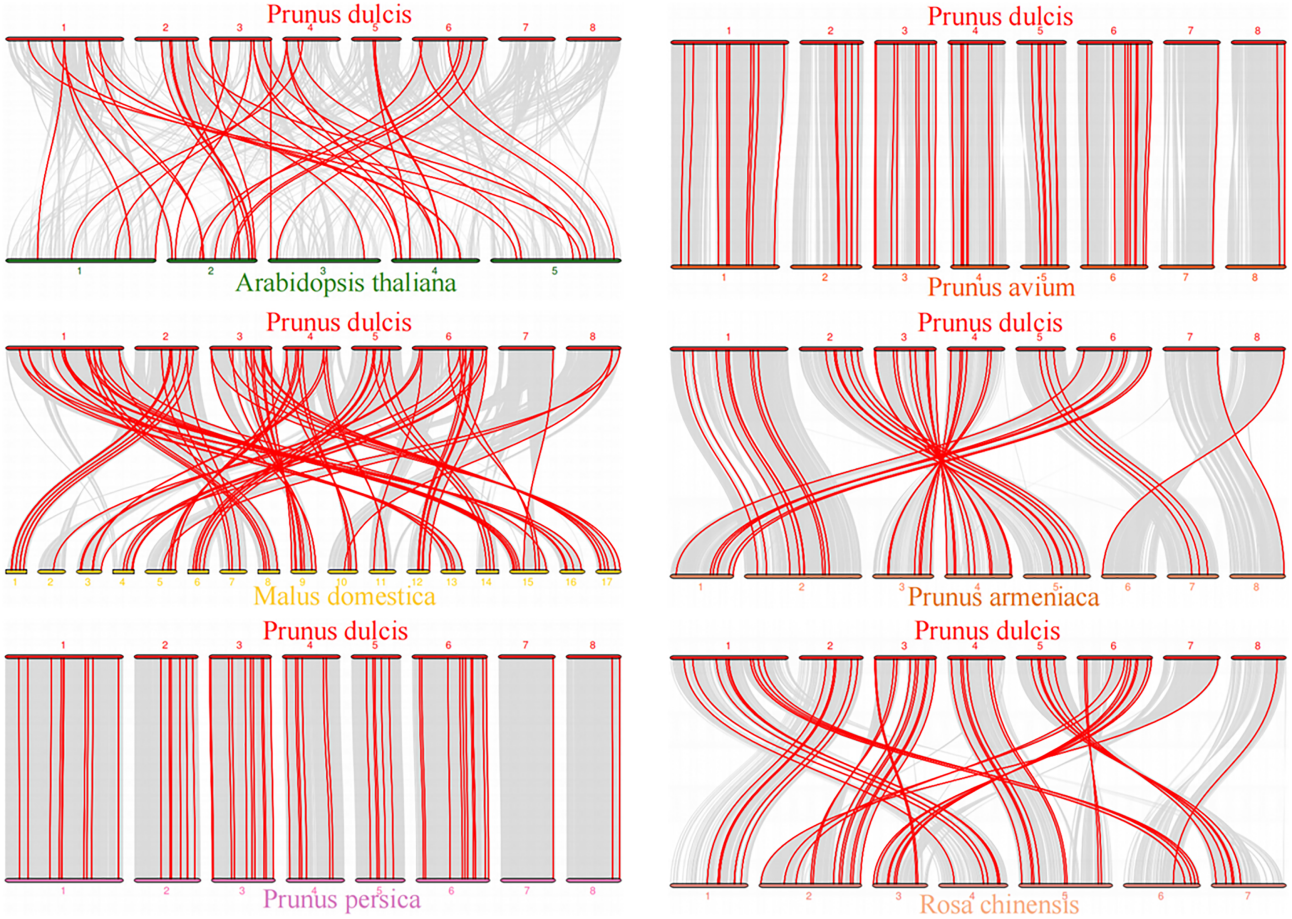
*Prunus dulcis* and *Arabidopsis thaliana*, *Malus domestica*, *Prunus persica*, *Prunus avium*, *Prunus armeniaca* and *Rosa chinensis* collinearity gene map. The syntenic *WRKY* gene pairs between almond and other species were highlighted with the red lines.

To explore the evolutionary relationship of the *PdWRKY* gene family members, we constructed a comparative collinear map of six species to depict the collinearity of *WRKY* gene members in almond, *Arabidopsis thaliana* and five representative *Rosaceae* species (Fig. 5; Table S5). The results showed that *Prunus dulcis* had 38, 82, 55, 54, 46 and 51 collinear gene pairs with *Arabidopsis thaliana*, *Malus domestica*, *Prunus persica*, *Prunus avium*, *Prunus armeniaca* and *Rosa chinensis*, respectively. Compared with *Arabidopsis thaliana*, *Prunus dulcis* and *Rosaceae* have more collinear *WRKY* genes.

**Figure 6:**
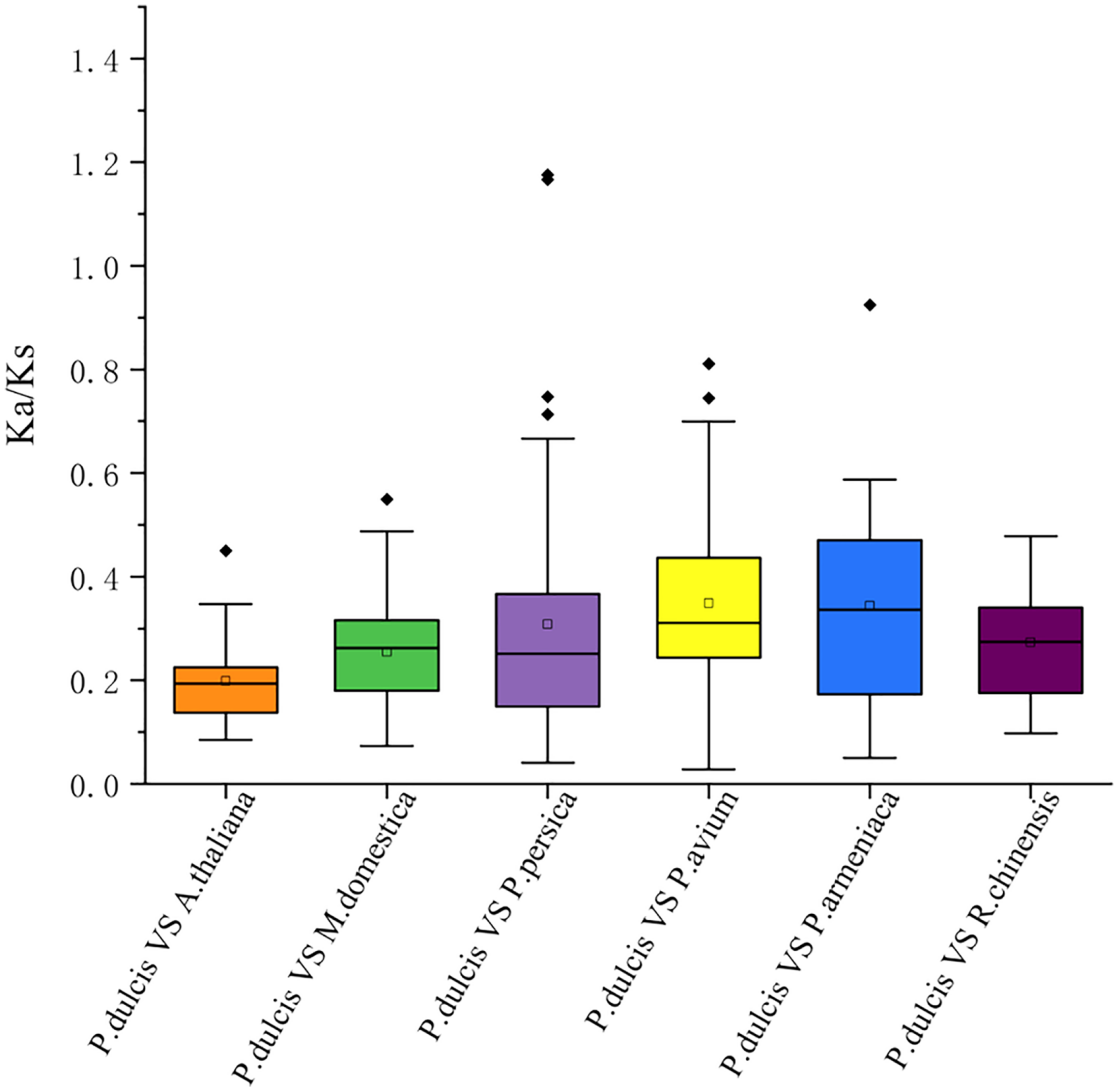
The ratio of nonsynonymous to synonymous substitutions (Ka/Ks) of *WRKY* genes in almond and other six species. The species’ names with the prefixes ‘*P. dulcis*’, ‘*A. thaliana*’, ‘*M. domestica*’, ‘*P. persica*’, ‘*P. avium*’, ‘*P. armeniaca*’ and ‘*R. chinensis*’ indicated *Prunus dulcis*, *Arabidopsis thaliana*, *Malus domestica*, *Prunus persica*, *Prunus avium*, *Prunus armeniaca* and *Rosa chinensis*, respectively.

This study further calculated the Ka/Ks ratios between the *PdWRKY* duplications and collinear *WRKY* genes in almond and six other species to explore the *PdWRKY* gene family and *WRKY* gene evolution among species under selection. The results showed Ka/Ks ratios <1 for all duplicated *PdWRKY* genes and Ka/Ks ratios <1 for collinear *WRKY* genes among all species. Thus, the *PdWRKY* gene family members and *WRKY* genes in different species have experienced strong purifying selection during the evolution process (Fig. 6; Table S6).

### Upstream *cis*-regulatory elements

The promoter sequences 2,000-bp upstream of the *PdWRKY* genes were extracted for *cis*acting element analysis, and the numbers of target elements were counted (Fig. S2; Tables S7 and S8). The results showed that in addition to a large number of basic light, TATA- and CAAT-box elements, additional annotated elements were related to plant hormones, stress resistance, defence and stress reactions, drought induction, anaerobic induction, and growth and development, including the auxin, gibberellin, abscisic acid, and MeJA pathways, indicating that *PdWRKY* genes are widely involved in regulating a variety of life processes such as almond growth, development, metabolism and stress resistance.

### Expression pattern analysis of the *PdWRKY* family members

**Figure 7:**
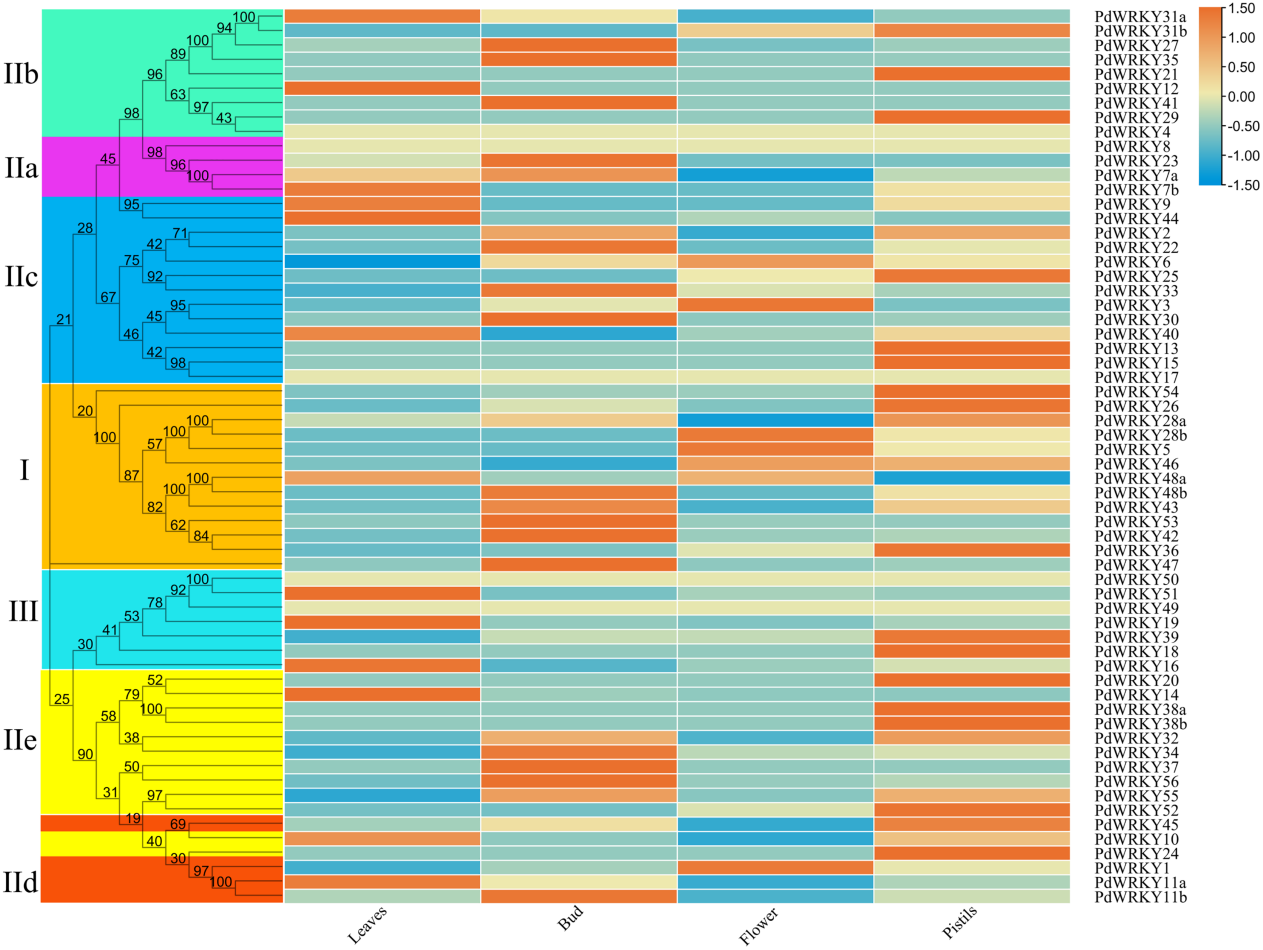
Expression patterns of *PdWRKY* genes in leaves, buds, flowers and pistils during almond development. The ROW normalization method was used to draw the heatmap. Red squares indicate up-regulation of expression, white square means no expression, green squares indicate down-regulated expression.

The transcriptome data for almond leaves, buds, flowers and pistils were selected for expression analysis across different tissues. The results showed that five *PdWRKY* genes were not expressed in these four tissues (Fig. 7; Table S9). These genes may be pseudogenes or genes with special temporal and spatial expression, or they may be expressed in other tissues. The other 57 *PdWRKY* genes were expressed in at least one tissue, and most of the *PdWRKY* genes were only expressed at low levels. This result may be related to the interaction of WRKY transcription factors with other genes or proteins during plant growth and development. There were 13, 20 and 22 *PdWRKY* genes expressed in leaves, buds and pistils, respectively, with at least one member of each group expressed in each tissue. In particular, the FPKM value of *PdWRKY53* in buds was as high as 153.85. This finding indicates that this gene may play an important role in the development of almond buds. In addition, flowers expressed only seven *PdWRKY* genes as follows: Group I (four), Group IIc (two) and Group IId (one). Notably, 57 *PdWRKYs* were expressed in different tissues, indicating that *PdWRKY* genes have significant tissue-specific expression characteristics.

### Expression analysis of *PdWRKY* family members

**Figure 8:**
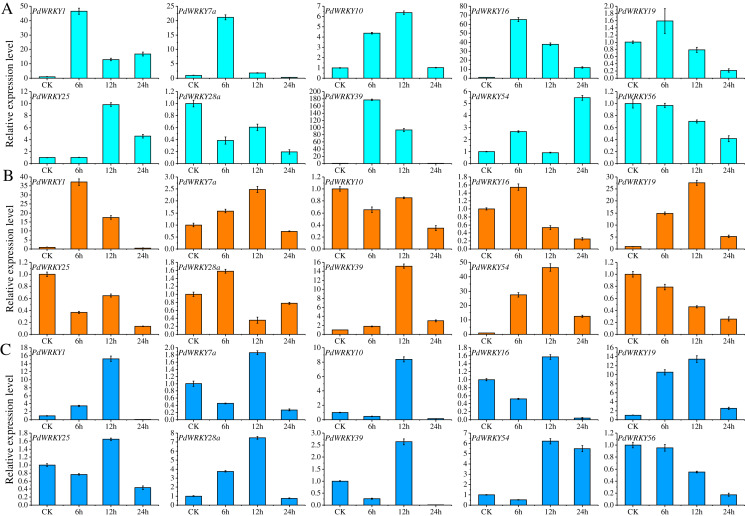
Analysis of relative expression of 10 *PdWRKY* genes in leaf tissue. (A) Low-temperature stress. (B) Drought stress. (C) Salt stress.

Ten *PdWRKY* genes were selected for qRT–PCR analysis to determine their expression levels in almond leaves at four time points, 0 (CK), 6, 12 and 24 h, under low-temperature, drought and salt stress (Fig. 8). After 6 h of low-temperature stress, five genes, *PdWRKY1/7a/10/16/39*, were highly expressed, especially *PdWRKY39*. At 12 h, only the expression of *PdWRKY10* and *PdWRKY25* was increased, while that of the other genes decreased, and at 24 h, four genes, *PdWRKY1/16/25/54*, were expressed at higher levels. When drought stress was applied for 6 h, only three genes, *PdWRKY1/19/54*, were highly expressed; at 12 h, four genes *PdWRKY1/19/39/54*, were highly expressed; and at 24 h, the expression of all examined genes was inhibited. After 6 h of salt stress treatment, only *PdWRKY19* was highly expressed; at 12 h, three genes, *PdWRKY1/10/19*, were highly expressed; and at 24 h, the expression of all genes was inhibited. The results show that the members of the *PdWRKY* family have different temporal expression characteristics under different stresses.

### PdWRKY protein interactions and GO annotations

**Figure 9:**
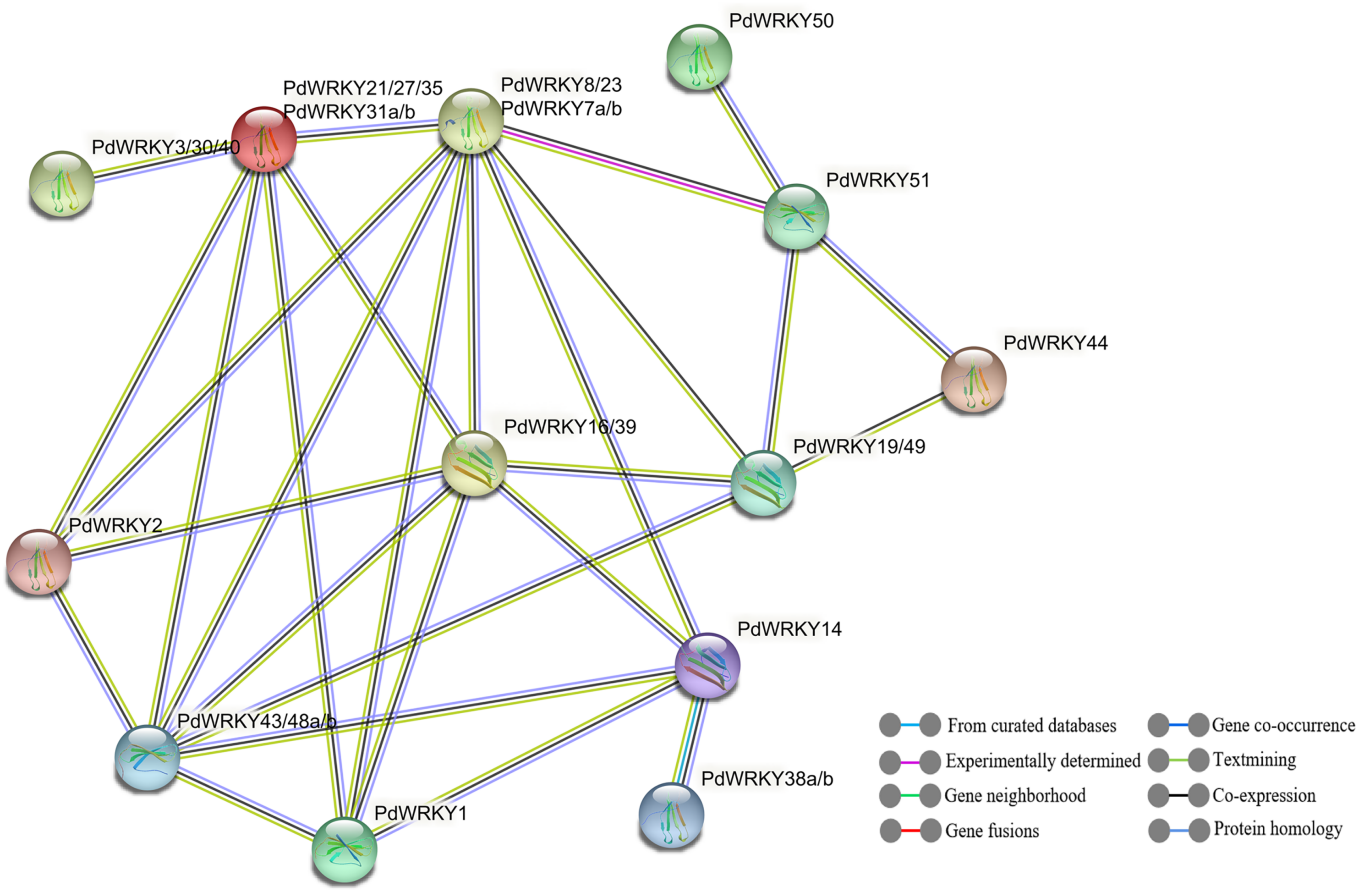
PdWRKY members protein–protein interaction network.

The STRING database was used to predict potential interactions between PdWRKY proteins (Fig. 9). In the PdWRKY protein interaction network, 27 members connected to each other to form 13 nodes; some nodes have multiple PdWRKY members, and each node interacts with other nodes. Some proteins exhibited direct interactions, such as PdWRKY51 and PdWRKY44, while others exhibited more complex polygenic interactions, such as PdWRKY16/39, PdWRKY19/49, and PdWRKY43/48a/48b. Notably, multiple members, such as PdWRKY43/48a/48b and PdWRKY7a/7b/8/23, were predicted as central nodes and were connected to multiple genes. In addition, GO annotation and enrichment analyses of PdWRKY proteins were performed (Table S10). The results showed that nine binding-related protein functions were annotated in the biological process category, such as nucleic acid binding, DNA binding and organic cyclic compound binding. Forty-eight functions were enriched in the molecular function category and were mainly related to metabolism and gene expression, such as nitrogen compound metabolic process, biosynthetic process and gene expression.

### Identification and annotation of *PdWRKY* target genes

**Figure 10:**
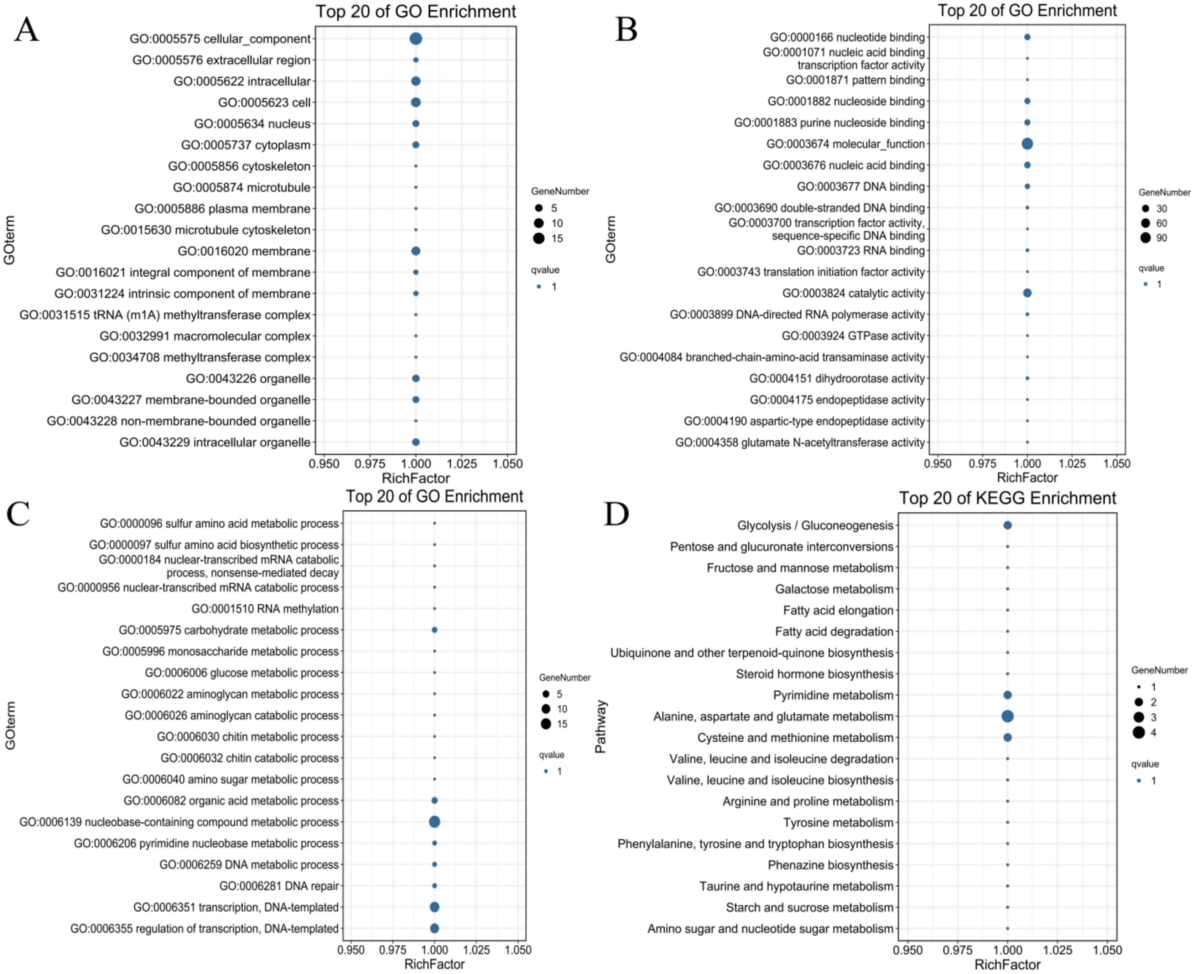
The top 20 GO terms and KEGG pathways enriched of candidate *PdWRKY* target genes. (A, B, C) The GO items Cellular Component, Molecular Function, and Biological Process, respectively; (D) the KEGG pathway.

To identify the possible downstream target genes regulated by the *PdWRKYs* and determine their functions, we searched the promoter sequence 2,000-bp upstream of the *PdWRKYs* using the conserved WRKY motif in the JASPAR database (Fig. S3). A total of 321 target genes were identified. Of these 321 genes, 128 had GO annotations, and 100 were mapped to the KEGG database (Table S11). GO analysis showed that multiple protein functions, such as metabolic process (GO:0008152), catalytic activity (GO:0003824) and catalytic activity (GO:0003824), were highly enriched in the target genes. The top 20 GO terms with the most target gene enrichment are shown in Fig. 10. In the cellular component category, most annotations were related to cell and membrane functions, including the terms cell (GO:0005623), intracellular (GO:0005622) and membrane (GO:0016020) (Fig. 10A). Binding (GO:0005488) function was the most enriched among the molecular function categories (Fig. 10B), while metabolic process (GO:0008152) was the most enriched among the biological process categories (Fig. 10C). Similarly, in the KEGG analysis, 86 target genes were annotated into metabolism-related pathways (Fig. 10D). The top 20 KEGG pathways for target gene enrichment included glycolysis/gluconeogenesis (ko00010), pentose and glucuronate interconversions (ko00040), fructose and mannose metabolism (ko00051), and alanine, aspartate and glutamate metabolism (ko00250). These results suggest that PdWRKY can affect multiple pathways by regulating target genes. In addition, more than 100 types of protein domains were found for the target genes, such as ribonucleases, UDP-glycosyltransferases and zinc fingers, indicating that WRKYs regulate a wide range of target genes to affect the growth and development of almond.

## Discussion

Almond (*Prunus dulcis*) is an important dry fruit trees with a wide distribution (Gharaghani, Solhjoo & Oraguzie, 2017). Almond are consumed worldwide, and almond tree are considered an economically important fruit tree in Xinjiang, China, where it is known as the ‘sacred fruit’. There are many almond varieties, and their self-incompatibility promotes genetic recombination between different varieties. Among the plant gene families, the WRKY transcription factors family is one of the largest and most important. The WRKY family has a wide range of roles in regulating plant growth and development, signal transduction, and stress response (Wani et al., 2021). Due to its importance, the *PdWRKY* gene family was identified based on the whole genome of *Prunus dulcis*.

In this study, a total of 62 *PdWRKY* genes were screened from the almond genome, which was similar to the finding of 58 *PpWRKY* genes in peaches (Chen et al., 2016). Based on the phylogenetic tree, the PdWRKY and AtWRKY proteins were clustered into three groups (I, II, and III) and five subgroups (IIa, IIb, IIc, IId, and IIe), among which the members of Group I have two WRKY structural domains. This result is consistent with that observed in *Arabidopsis thaliana* (Wei et al., 2012). In addition, according to the *WRKY* genes found in different groups, it is speculated that the *WRKY* genes in Group I may have a longer evolutionary time than those in the other groups. The *PdWRKY* genes in Groups I, II, and III accounted for 19.35%, 69.35%, and 11.29% of the total *PdWRKY* genes, respectively, which is consistent with previous results obtained for *WRKY* gene family members in various dicotyledonous plants, such as *Arabidopsis thaliana* and peach (Bao et al., 2019). Losses or changes in gene domains are more common in monocots than in dicots and may have led to the expansion of *WRKY* gene family domains (Wei et al., 2012). The multiple alignment results for the PdWRKY proteins showed that the C-terminus has a conserved C2H2 or C2HC zinc finger structure and that the N-terminus has a highly conserved heptapeptide sequence (WRKYGQK). In addition, in PdWRKY9 and PdWRKY44, the heptapeptide sequence WRKYGQK has been changed to WRKYGKK, and this change is also found in *Arabidopsis thaliana*, apple and potato.

Gene family evolution and phylogenetic classification can be assessed based on the structural diversity of genes (Hu et al., 2010). Thirty *PdWRKY* (48.39%) genes have three exons or two introns, which is consistent with the three exons in the *WRKY* gene family in peach (50.00%), pear (57.12%) and cassava (49.41%). In addition, we identified a total of 10 conserved motifs (ranging in length from 11 to 41 amino acid residues) in 62 PdWRKYs. PdWRKY proteins within the same group had a highly conserved motif type, which means that the *PdWRKY* genes in the same group may have been highly conserved during the process of evolution. In conclusion, genes in the same group have more conserved motif types and gene structures.

Gene duplication is the main factor leading to the rapid expansion and evolution of gene families (Jouffrey, Leonard & Ahnert, 2021). Studies have shown that the *Arabidopsis thaliana* Group III *WRKY* gene family rapidly expanded through tandem duplication (Zhang & Wang, 2005). In Group III, two gene pairs, *PdWRKY49/PdWRKY50* and *PdWRKY50/PdWRKY51*, are tandemly duplicated. Sixty-two *PdWRKY* members contain 24 pairs of segmental duplications and nine pairs of tandem duplications, indicating that segmental duplications are the main amplification mode in *PdWRKY* members, which is consistent with the amplification mode of *WRKY* family members in many other plants, such as peach, maize and rice. In addition, *Prunus dulcis* shared 38, 82, 55, 54, 46 and 51 pairs of collinear genes with *Arabidopsis thaliana*, *Malus domestica*, *Prunus persica*, *Prunus avium*, *Prunus armeniaca* and *Rosa chinensis*, respectively. In particular, *Prunus dulcis* has multiple genes corresponding to one *Malus domestica WRKY* gene, and four *WRKY* genes in *Prunus persica*, *Prunus avium*, *Prunus armeniaca* and *Rosa chinensis* have one-to-one relationships. This result further shows that *WRKY* genes have been highly conserved based on homology during the evolution of *Rosaceae*. Exploring the Ka/Ks ratios of duplicated genes is an effective way to study the effects of duplicated genes on evolution. The Ka/Ks ratios of 21 pairs of segmental duplications and five pairs of tandem duplications in the *PdWRKY* members were all less than 1, indicating that these genes were mainly affected by purifying selection. The Ka/Ks ratios of *PdWRKY* members in almond and the collinear gene pairs from six other species were all less than 1, indicating that the *WRKY* genes were also subject to purifying selection during the evolution of different species. *Cis-*elements play an important role in the transcriptional regulation of gene expression (Wang et al., 2017). In this study, we analysed the *cis*-acting elements in the 2,000-bp promoter regions upstream of the *PdWRKY* genes. A variety of elements related to plant growth and development were detected, such as *cis*-acting elements involved in cell cycle regulation and auxin-responsive elements, as well as *cis*-acting regulatory elements related to meristem expression. We also detected cis elements related to abiotic stress, such as *cis*-acting elements involved in defence and stress responses, *cis*-acting elements involved in low-temperature responses, and myb binding sites involved in drop ability. Therefore, *cis*-acting element analysis provides clues for the functional study of *PdWRKY* members, especially for the regulation of related genes and plant development under different stresses.

Gene expression patterns can provide basic information for determining the biological functions of genes (Xu et al., 2015). The expression pattern results showed that the expression levels of five genes, *PdWRKY4/8/17/49/50*, in the four tissues were all 0. Notably, the other 57 genes were only significantly expressed in one tissue. In addition, the FPKM values of the PdWRKY members were generally low, which may be related to the frequent combination of *WRKY* gene transcriptional regulation with downstream target genes involved in various physiological and developmental pathways. Studies on peach, apricot, and plum have found that *WRKY* genes are involved in the bud dormancy process.

Related studies have shown that *WRKY* genes are involved in responses to abiotic stresses, such as low-temperature, drought and salt stresses, in many plants (Jiang et al., 2017). *PmWRKY18/27/55* (*Prunus mume*) and *PpWRKY11/33/48* (*Prunus persica*) showed different degrees of response to low-temperature stress (Jiang et al., 2017; Chen et al., 2016), and *CsWRKY3/7/35* (*Camellia sinensis*) increased significantly under low-temperature, drought and salt stresses (Wang et al., 2019). According to the qRT–PCR results, the expression of 10 *PdWRKY* genes changed at different time points (0, 6, 12, and 24 h) under low-temperature, drought and salt stress in almond leaves, showing the characteristics of temporal and spatial expression changes. Five genes, *PdWRKY1/7a/10/16/39*, all had high expression levels under low-temperature stress at 6 h. The expression of these genes decreased over time, especially at 24 h, and the expression of most of these genes was suppressed. Under drought stress, two genes, *PdWRKY19* and *PdWRKY54*, had higher expression levels at all time points, indicating that these two genes are mainly involved in the resistance of almond to the effects of drought stress. Under salt stress, *PdWRKY19* had a higher expression level at all time points, while the expression of the other genes was inhibited at 24 h. In general, some *PdWRKY* genes in almond were involved in resistance to low-temperature, drought and salt stresses, but their expression levels were all high before 12 h and decreased over time after 12 h. Especially under low-temperature stresses, the expression of multiple genes is very significant and may be related to the temperature of the almond cultivation area in Xinjiang. In this area, almond often encounters the influence of cold currents and low temperatures in the spring and thus exhibits increased resistance to low-temperature stress (Bao et al., 2019).

## Conclusion

In this study, 62 *PdWRKY* genes were identified from the almond genome. Phylogenetic, gene structures, chromosome location and promoter analyses were used to provide complete information about the *WRKY* gene family in almond. Segmental duplication is the main amplification pathway in the *WRKY* gene family in almond, and the Ka/Ks ratios of collinear genes indicate that these genes are mainly affected by purifying selection. Additionally, the qRT–PCR results show that *PdWRKY* genes are involved in the resistance of almond to the effects of low-temperature, drought and salt stresses. In conclusion, this study provides a good basis for further studies of the biological functions and evolution of the *PdWRKY* genes family.

## Supplemental Information

10.7717/peerj.13491/supp-1 Supplemental Information 1 Multiple sequence alignment of PdWRKY proteins. The red areas represent highly conserved sequences.

10.7717/peerj.13491/supp-2 Supplemental Information 2 *PdWRKY* family members *cis*-acting elements.

10.7717/peerj.13491/supp-3 Supplemental Information 3 The consensus motif of the WRKY DNA binding site from the JASPA_CORE database.

10.7717/peerj.13491/supp-4 Supplemental Information 4 Sequences of primers for qRT-PCR.

10.7717/peerj.13491/supp-5 Supplemental Information 5 Quantitative expression of gene fluorescence.

10.7717/peerj.13491/supp-6 Supplemental Information 6 Characteristics of *WRKY* gene family members in almond genome. The sequences of *WRKY* proteins in almond

10.7717/peerj.13491/supp-7 Supplemental Information 7 Characteristics of *WRKY* gene family members in almond genome.

10.7717/peerj.13491/supp-8 Supplemental Information 8 Orthologous relationships of *WRKY* genes between almond and other six plant species.

10.7717/peerj.13491/supp-9 Supplemental Information 9 *WRKY* genes replication and homologous gene Ka/Ks values in almond.

10.7717/peerj.13491/supp-10 Supplemental Information 10 *Cis*-element analyses of the *PdWRKY* genes.

10.7717/peerj.13491/supp-11 Supplemental Information 11 *Cis*-element analysis of 2000 bp nucleotide sequences data upstream of the translation initiation codon of *PdWRKY* genes.

10.7717/peerj.13491/supp-12 Supplemental Information 12 Expression profiles of *PdWRKY* genes in multiple tissues.

10.7717/peerj.13491/supp-13 Supplemental Information 13 Functional annotation of GO enrichment of *WRKY* proteins in almond.

10.7717/peerj.13491/supp-14 Supplemental Information 14 Prediction and analysis of potential *PdWRKY* target genes.

## Additional Information and Declarations

### Data Availability

The following information was supplied regarding data availability: The raw measurements are available in the Supplemental Files.
