# Supplementary figures and images for: Characterization of the WRKY gene family reveals its contribution to the adaptability of almond (Prunus dulcis)
Source: PeerJ. 2022 Jul 4;10:e13491. doi: 10.7717/peerj.13491 (PMC9261925; doi:10.7717/peerj.13491)

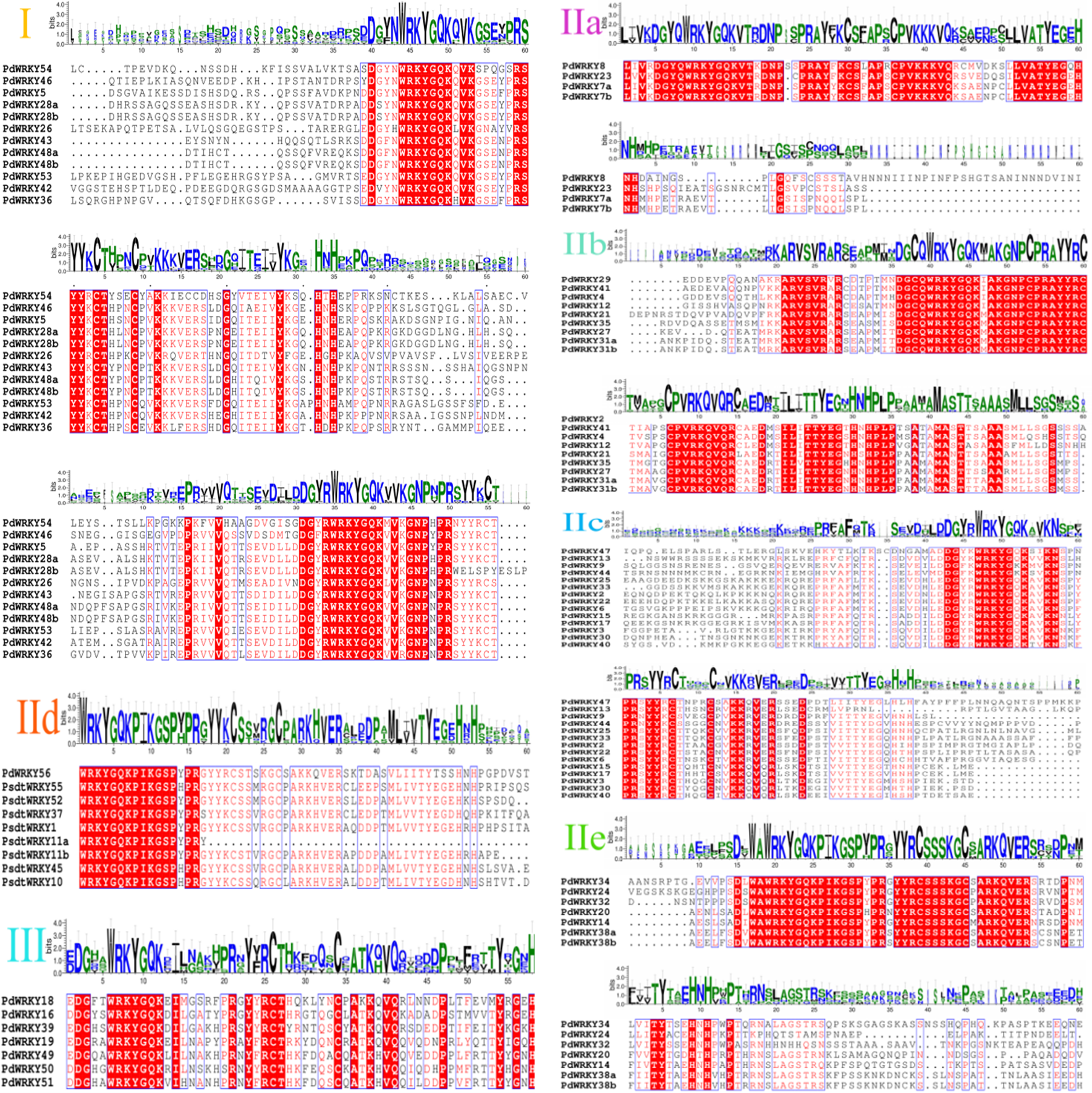

Supplement: Supplemental Information 1 — The red areas represent highly conserved sequences. [file peerj-10-13491-s001.png]

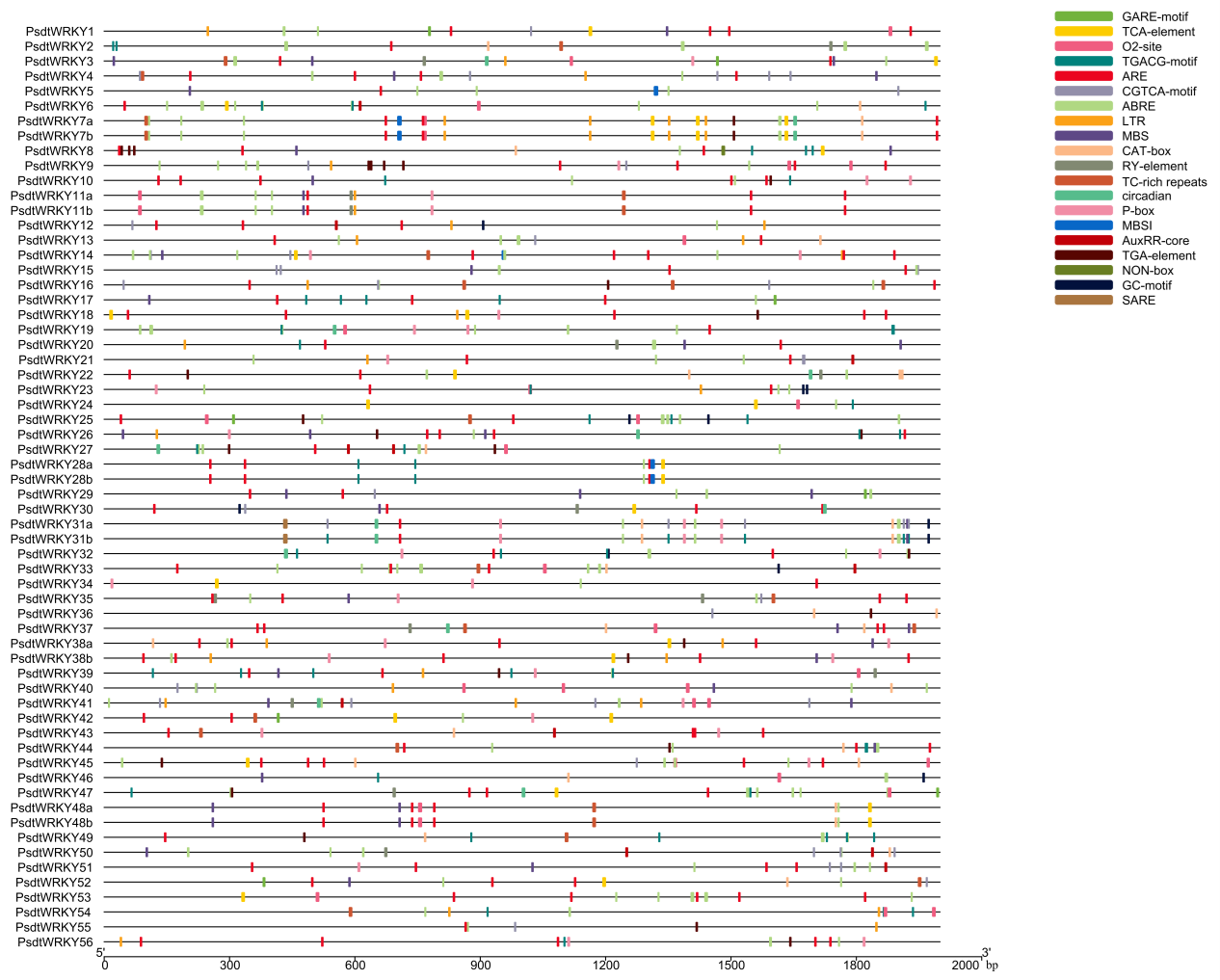

Supplement: Supplemental Information 2 [file peerj-10-13491-s002.png]

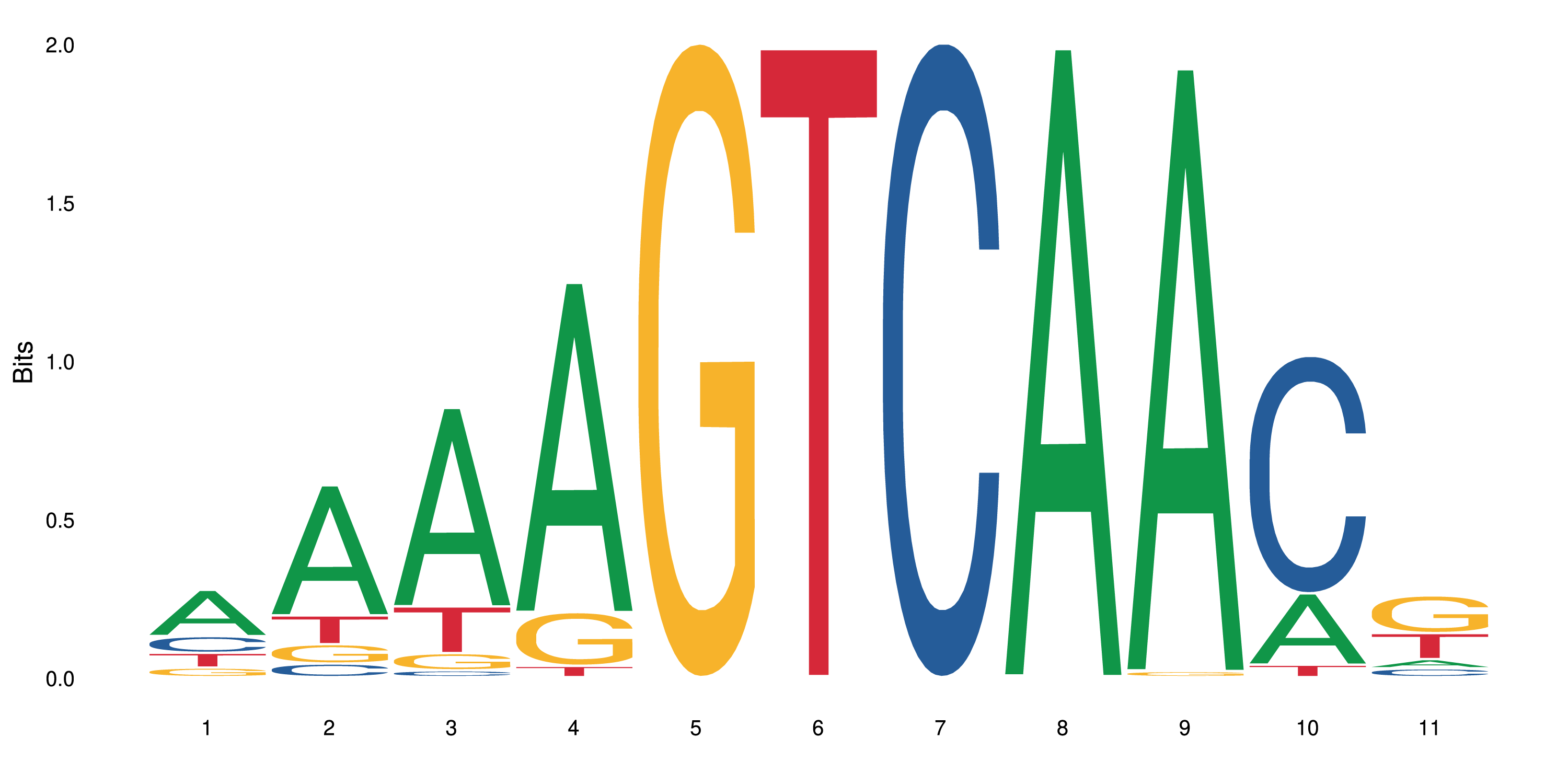

Supplement: Supplemental Information 3 [file peerj-10-13491-s003.png]
